# Molecular Network Approach Reveals *Rictor* as a Central Target of Cardiac ProtectomiRs

**DOI:** 10.3390/ijms22179539

**Published:** 2021-09-02

**Authors:** András Makkos, Bence Ágg, Zoltán V. Varga, Zoltán Giricz, Mariann Gyöngyösi, Dominika Lukovic, Rainer Schulz, Monika Barteková, Anikó Görbe, Péter Ferdinandy

**Affiliations:** 1 Cardiovascular and Metabolic Research Group, Department of Pharmacology and Pharmacotherapy, Semmelweis University, 1089 Budapest, Hungary; makkos.andras@med.semmelweis-univ.hu (A.M.); agg.bence@med.semmelweis-univ.hu (B.Á.); varga.zoltan@med.semmelweis-univ.hu (Z.V.V.); giricz.zoltan@med.semmelweis-univ.hu (Z.G.); peter.ferdinandy@pharmahungary.com (P.F.); 2 MTA-SE System Pharmacology Research Group, Department of Pharmacology and Pharmacotherapy, Semmelweis University, 1089 Budapest, Hungary; 3 Pharmahungary Group, 6722 Szeged, Hungary; 4 HCEMM-SU Cardiometabolic Immunology Research Group, Department of Pharmacology and Pharmacotherapy, Semmelweis University, 1089 Budapest, Hungary; 5 Division of Cardiology, Medical University of Vienna, 1090 Vienna, Austria; mariann.gyongyosi@meduniwien.ac.at (M.G.); dominika.lukovic@meduniwien.ac.at (D.L.); 6 Institute of Physiology, Justus Liebig University Giessen, 35392 Giessen, Germany; rainer.schulz@physiologie.med.uni-giessen.de; 7 Institute for Heart Research, Centre of Experimental Medicine, Slovak Academy of Sciences, Dúbravská cesta 9, 84104 Bratislava, Slovakia; monika.bartekova@savba.sk; 8 Institute of Physiology, Comenius University in Bratislava, 81108 Bratislava, Slovakia

**Keywords:** network theory, cardioprotection, vasculoprotection, microRNA, miRNA, *Rictor*

## Abstract

Cardioprotective medications are still unmet clinical needs. We have previously identified several cardioprotective microRNAs (termed ProtectomiRs), the mRNA targets of which may reveal new drug targets for cardioprotection. Here we aimed to identify key molecular targets of ProtectomiRs and confirm their association with cardioprotection in a translational pig model of acute myocardial infarction (AMI). By using a network theoretical approach, we identified 882 potential target genes of 18 previously identified protectomiRs. The *Rictor* gene was the most central and it was ranked first in the protectomiR-target mRNA molecular network with the highest node degree of 5. Therefore, *Rictor* and its targeting microRNAs were further validated in heart samples obtained from a translational pig model of AMI and cardioprotection induced by pre- or postconditioning. Three out of five *Rictor*-targeting pig homologue of rat ProtectomiRs showed significant upregulation in postconditioned but not in preconditioned pig hearts. *Rictor* was downregulated at the mRNA and protein level in ischemic postconditioning but not in ischemic preconditioning. This is the first demonstration that *Rictor* is the central molecular target of ProtectomiRs and that decreased *Rictor* expression may regulate ischemic postconditioning-, but not preconditioning-induced acute cardioprotection. We conclude that *Rictor* is a potential novel drug target for acute cardioprotection.

## 1. Introduction

Protection of the ischemic heart is an unmet clinical need, as cardiovascular morbidity and mortality are exponentially increasing worldwide. There are several promising targets for cardioprotection, which were successfully tested in preclinical in vitro and small animal models. However, their translation to the clinical practice as treatment of acute myocardial infarction (AMI) has failed so far [1,2]. Therefore, effective cardioprotective therapy is still not available. For finding new cardioprotective targets, the application of unbiased search for players in cardioprotection, such as microRNAs and their target mRNAs using network analysis, may lead to finding optimal targets and more effective translation.

The importance of microRNAs in cardiac pathologies and in cardioprotection suggests their promising therapeutic potential [3,4]. We have previously identified several cardioprotective candidate microRNAs termed ProtectomiRs by a systemic analysis of microRNA expression pattern in cardioprotection induced by ischemic pre- and postconditioning as compared to ischemia-reperfusion injury. Additionally, the cytoprotective effects of some ProtectomiR candidates (miR-139-5p, miR-125b*, let-7b and miR-487b) were validated in isolated cardiac myocytes [5]. Modulation of microRNA expression could be a feasible therapeutic approach, both with microRNA inhibitors (antagomiRs) and microRNA mimics [3,6]. MicroRNAs can regulate the expression of multiple genes and each gene can be regulated by multiple microRNAs. The combination of additive or synergistic effects of a multitarget therapy may provide optimal cardioprotection [7]. Target prediction by microRNA-target interactome network analysis and experimental target validation is an emerging unbiased approach to better understand the pathomechanisms and to identify new potential molecular targets for therapy [3,8,9,10,11].

Target validation in large animals has high translational value, especially application of porcine animal models of cardiovascular diseases, including AMI [1,12]. In porcine AMI models, clinically relevant endpoints have been established [13], which allow correlation of preclinical outcome with future clinical examinations [14,15]; moreover, tissue samples can be used for cellular or molecular analysis [16].

In the present study, we aimed to identify key molecular targets of ProtectomiRs found in a previous rat study and validate their targets using tissue samples from a clinically relevant pig model of AMI and cardioprotection by ischemic pre- and postconditioning.

## 2. Results

### 2.1. microRNA Target Prediction and ProtectomiR microRNA-mRNA Target Interaction Network

**Figure 1:**
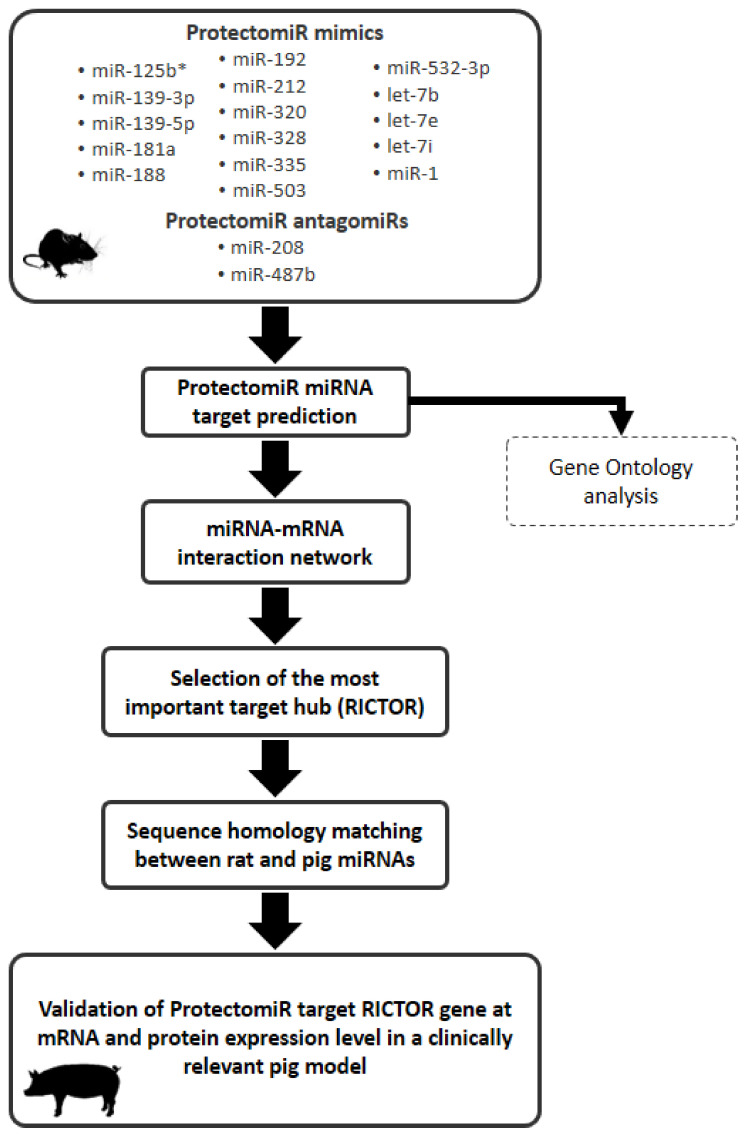
Experimental protocol.

**Figure 2:**
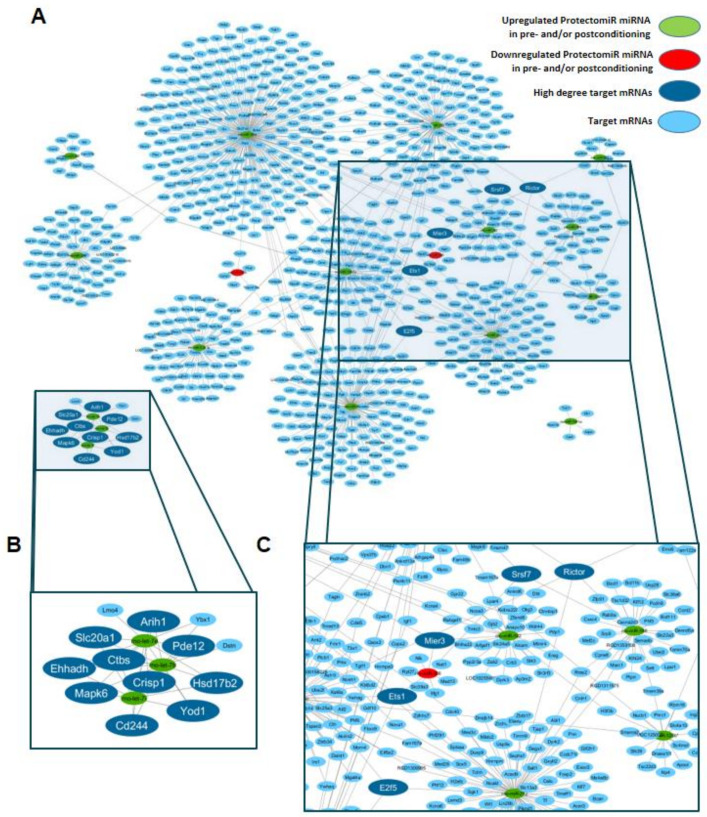
Predicted interaction network of ProtectomiR microRNAs (**A**) showing the network neighborhood of predicted hub genes with at least three microRNA–mRNA target interactions (node degree) (**B**,**C**). Panel (C) shows the role of the most central target hub *Rictor*. MicroRNAs upregulated and downregulated in pre- and/or postconditioning and target mRNAs are indicated in green, red and blue, respectively. Dark blue nodes represent mRNAs with a node degree of at least 3. A high-resolution version of the network is available as Supplementary Figure S1.

Eighteen different protectomiRs identified in our previous rat myocardial infarction and cardioprotection model (Figure 1) revealed 882 predicted target mRNAs by in silico target prediction. The microRNA–mRNA interactions were visualized to highlight the central hub of the mRNA targets (Figure 2). In this interaction network, 84 mRNAs had interactions with more than one microRNA (Supplementary Table S1), and 15 mRNAs interacted with at least three microRNAs.

### 2.2. Selection of the Most Important Target Hub

**Figure 3:**
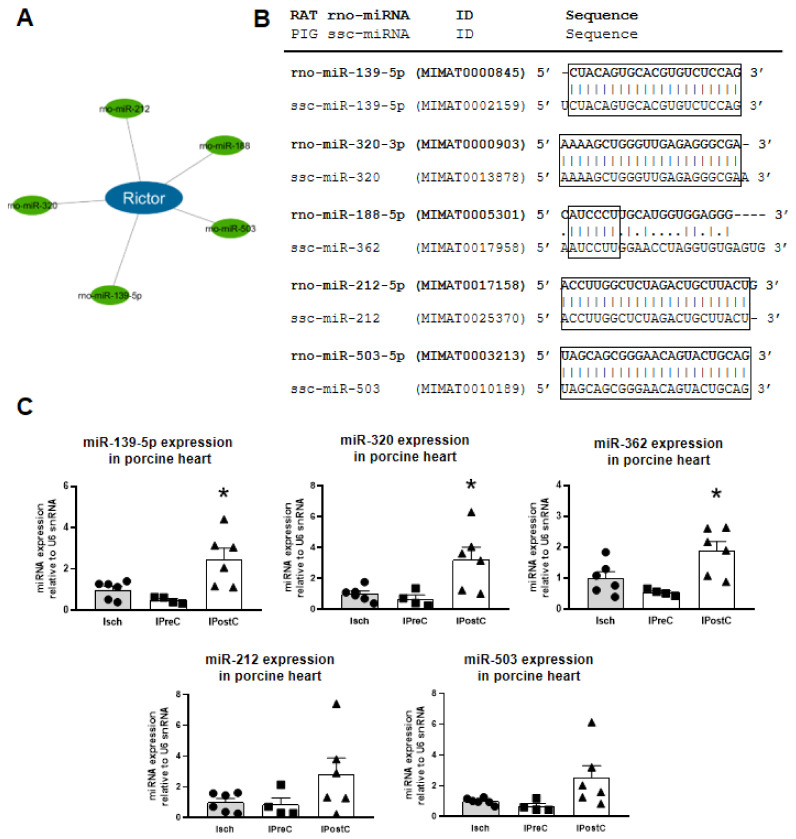
Pig homologues of the *Rictor* targeting microRNAs (**A**) were identified based on rat–pig microRNA sequence similarity. Four of the five rat microRNAs (miR-139-5p, miR-320, miR-212, miR-503) showed a 100% match and one (rno-miR-188-5p vs miR-362) showed a 56% match between pig and rat microRNA homologues (**B**). Expression levels of the five *Rictor* targeting ProtectomiR microRNA homologues were tested with qPCR in ischemic preconditioning and postconditioning compared to ischemia-reperfusion (**C**). Black circle, triangle and square signs represent individual data points. * *p* < 0.05 vs. Isch group, *n* = 4–6, one-way ANOVA followed by Dunnett post-hoc test.

The *Rictor* gene was identified as the most central target hub with the highest node degree, interacting with five different microRNAs (miR-139-5p, miR-320, miR-212, miR-503, miR-188-5p) out of the 18 investigated protectomiRs (Table 1 and Figure 3A).

### 2.3. Rat–Pig microRNA Homology Matching

We used left myocardial tissue samples from a clinically relevant porcine AMI model to validate the central role of *Rictor* in ischemic pre- and postconditioning. Therefore, first we identified the pig homologues of the *Rictor*-targeting microRNAs based on rat–pig microRNA sequence similarity. Four of the five rat microRNAs (miR-139-5p, miR-320, miR-212, miR-503) showed a total sequence match between rat and pig microRNA homologues. In the case of rno-miR-188-5p rat microRNA we identified the scc-miR-362 with 50% homology. MiR-362 is a member of miR-188 microRNA family and it has an identical seed sequence to rno-miR-188-5p (Figure 3B).

### 2.4. Expression of Rictor-Targeting microRNAs in Myocardium of a Clinically Relevant Closed Chest Porcine Model

We found up-regulation of three *Rictor* gene-targeting microRNAs out of five targeting microRNAs in the interaction network in the IPostC group. Two other microRNAs showed a tendency but not a statistically significant change (Figure 3C). Interestingly, these microRNAs did not show alteration in the myocardial samples of the IPreC group.

### 2.5. Rictor mRNA Expression

**Figure 4:**
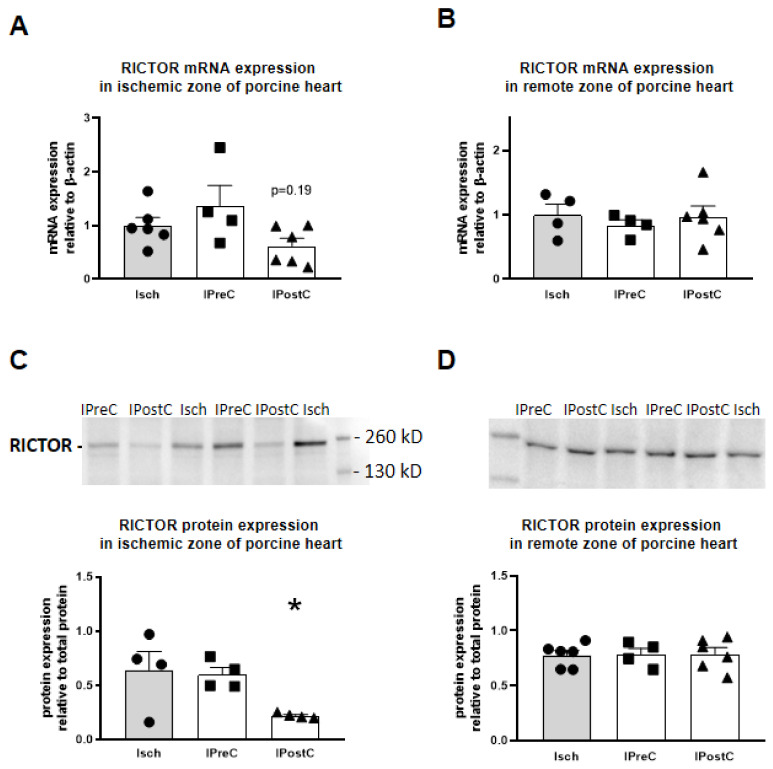
Central target hub, *Rictor* mRNA expression was measured with qPCR in the ischemic zone (**A**) and in the non-infarcted remote zone (**B**) of the porcine left ventricular myocardium. RICTOR protein expression was measured with Western blot in the ischemic zone (**C**) and in the non-infarcted remote zone (**D**) of the porcine left ventricular myocardium and normalized to total protein staining. Black circle, triangle and square signs represent individual data points. * *p* < 0.05 vs. Isch group, *n* = 4–6, one-way ANOVA followed by Dunnett post-hoc test.

mRNA expression of the *Rictor* gene was investigated in both the ischemic and non-ischemic zones of the porcine myocardium. We observed a statistically non-significant downregulation of *Rictor* mRNA in the ischemic zone of the postconditioned group (Figure 4A). There were no changes in *Rictor* mRNA expression in the IPreC group compared to Isch, neither in the ischemic nor in the non-ischemic (remote) myocardium zones (Figure 4A,B).

### 2.6. RICTOR Protein Expression

Protein expression of RICTOR was in line with the mRNA level expression changes. We observed a significant downregulation of the RICTOR protein in the ischemic zone of the IPostC group as compared to the Isch group (Figure 4C). No RICTOR protein expression changes were found in the IPreC group compared to Isch neither in the ischemic nor the non-ischemic (remote) myocardium zones (Figure 4C,D).

### 2.7. Phosphorylation Level of mTORC2 Downstream Mediators and Expression of Heat Shock Proteins in the Ischemic Zone of Myocardium

Neither the phosphorylation level of the protein kinase B (Akt), its downstream effector glycogen synthase kinase-3 (GSK-3) nor the protein kinase C (PKC) were significantly changed by either pre- or postconditioning. HSP70 or HSP90 also did not show a difference between experimental groups (Supplementary Figure S3).

### 2.8. Gene Ontology Enrichment Analysis of the Interaction Network

**Figure 5:**
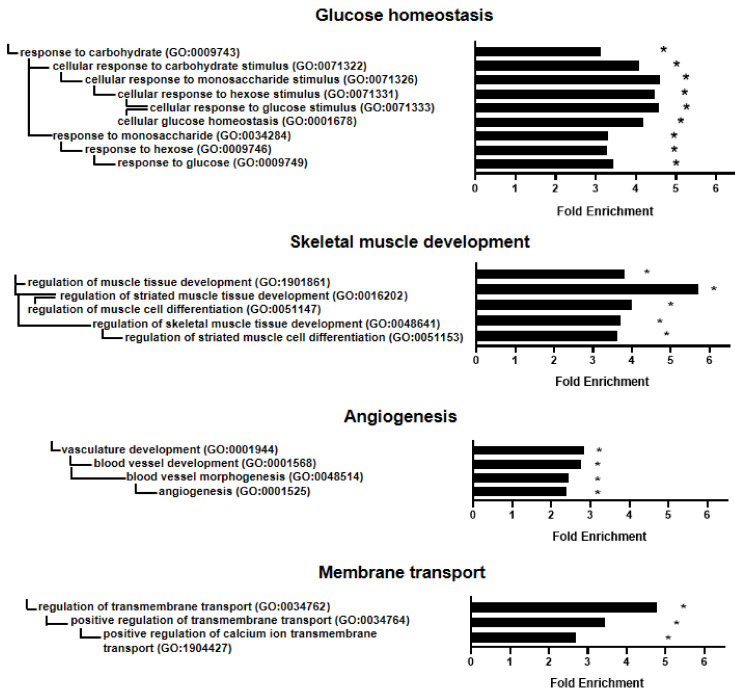
Gene ontology enrichment analysis (biological processes) of all microRNA target mRNAs (*n* = 882) highlights the effect of the ProtectomiR microRNAs on glucose homeostasis, skeletal muscle development, angiogenesis and membrane transport. A total of 21 biological processes out of the 25 with the highest fold enrichment value are presented here. * *p* < 0.05, vs. control (gene ontology enrichment analysis with Bonferroni correction).

To identify the most important biological processes mediated by target mRNAs of ProtectomiR microRNAs, Gene Ontology (GO) enrichment analysis was performed with the full list of predicted target mRNAs (882 predicted target genes) as an input (Supplementary Table S2). The top 25 significant hits of the GO analysis in the biological process category revealed mainly processes involved in glucose homeostasis, skeletal muscle development, angiogenesis and membrane transport (Figure 5).

## 3. Discussion

In the present study we analyzed a rat protectomiR mRNA molecular network in silico and found that the *Rictor* gene is the center of the network with the highest node degree. Furthermore, we experimentally validated the *Rictor* gene and its targeting microRNAs in the myocardium of a translational pig model of acute myocardial infarction and cardioprotection by pre- and postconditioning and found that the RICTOR protein was significantly decreased by ischemic postconditioning but not by preconditioning. This is the first demonstration that *Rictor* is the central molecular target of ProtectomiRs and that decreased *Rictor* expression may regulate ischemic postconditioning-, but not preconditioning-induced acute cardioprotection.

MicroRNAs, the small non-coding RNA molecules with inhibitory post-transcriptional effect, are in focus for translational medicine [3,17]. microRNAs are evolutionally conserved, which provides a great opportunity to identify their molecular targets (i.e., mRNAs) with high translational value [18]. We have previously identified 18 microRNAs with cardioprotective potential (termed protectomiRs) in a study of acute myocardial infarction and cardioprotection induced by ischemic pre- and postconditioning in rats by a systematic, unbiased microRNA expression pattern analysis [5]. In the present study, we predicted the molecular mRNA targets of the 18 potential ProtectomiRs in silico and revealed 882 potential target mRNAs. Here we utilized unbiased target prediction and network visualization for better understanding the mRNA targets regulated by the cardioprotection-associated ProtectomiRs according to the recommendations of the European Society of Cardiology [3]. For further selection, we ranked the targets according to the highest number of interactions and found 15 targets with at least three microRNA interactions. These targets represent the hubs of the interaction network we constructed here. These multiple targeted hubs could be classified into two subgroups. Ten of the hubs were organized around let-7b, let-7e and let-7i microRNAs, representing a smaller isolated subnetwork (e.g., *Arih1, Cd244, Crisp1, Ctbs, Ehhadh, Hsd17b2, Mapk6, Pde12, Slc20a1* and *Yod1*). Meanwhile, the other five multiple targeted hub genes (*Rictor, E2f5, Ets1, Srsf7* and *Mier3*) are part of a larger component of the network, which contains the majority of the predicted targets. Nevertheless, the present microRNA–mRNA target interaction network analysis revealed the *Rictor* gene as the highest degree hub with five interactions with protectomiRs. Therefore, in the present study we focused on the highest degree of *Rictor* gene expression, and have not further studied other high-degree mRNA targets.

The RICTOR protein is a key member of the mTORC2 protein complex, which regulates cell survival, proliferation, migration and cytoskeletal remodeling [19]. The fundamental role of mTORC2 activation in cell survival, in cardiac adaptation and in cardioprotection by ischemic preconditioning was shown in multiple previous publications [20,21]. We identified here the *Rictor* gene with the highest node degree, interacting with the miR-320, miR-188-5p, miR-139-5p, miR-212 and miR-503 protectomiRs that were upregulated in ischemic pre- and/or postconditioning in our previous studies in a rat myocardium [5]. Based on the antagonistic microRNA–target interactions, we predicted the downregulation of *Rictor* gene expression during cardioprotection by the upregulated targeting ProtectomiRs, which we measured at the mRNA and protein level.

Based on the above mentioned in silico results, we next experimentally validated the protectomiRs and their target *Rictor* in cardioprotection in a large animal model of cardioprotection after we confirmed that rat protectomiRs can be translated to pigs based on sequence homology. For experimental validation in the present study, we obtained cardiac tissue samples from our previous study in a translational pig model of acute myocardial infarction and cardioprotection by pre- and postconditioning where preconditioning mainly protected against infarct size, while postconditioning protected the microvasculature [13,22]. In the present study, three out of five mRNAs showed significant upregulation, while two showed non-significant tendencies of upregulation in the postconditioned pig myocardium, but none of the protectomiRs were changed in the preconditioned groups. Similarly, RICTOR protein expression was downregulated in the postconditioned but not in the preconditioned group. These results show that protectomiRs and their central target to inhibit *Rictor* expression may contribute to cardioprotection by ischemic postconditioning characterized mainly by microvascular protection as seen by decreased tissue edema and microvascular obstruction in this pig model [13,22]. However, it cannot be excluded that in contrast to preconditioning, where *Rictor* expression is intact, downregulation of the RICTOR protein in postconditioning may contribute to the observation that postconditioning does not decrease infarct size but show protection only on the microvasculature. The mechanism by which *Rictor* inhibition may contribute to microvascular protection in the ischemic heart is not known. Therefore, here we investigated two major signaling pathways downstream of the mTORC2 complex, Akt-GSK-3 and PKC [23], as well as heat shock proteins 70 and 90 that regulate the mTORC2 function [24]. Neither phosphorylation of Akt-GSK-3 and PKC nor HSP70 or HSP90 were significantly changed by either pre- or postconditioning. These results show that further studies are needed to clarify how downregulation of *Rictor* contributes to microvascular protection.

Moreover, other ProtectomiR target genes may also contribute to cardioprotection.

Therefore, we identified the effects of the whole ProtectomiR–mRNA target network on biological functions by Gene Ontology enrichment analysis. This showed that changes in glucose homeostasis, muscle development, angiogenesis and membrane transport mechanism may be involved in cardioprotection. Interestingly, the *Rictor* gene was not annotated in the altered GO terms; however, it has been shown in the literature that RICTOR and mTORC2 play a role in glucose homeostasis [25] and angiogenesis [26], showing obvious limitation in GO analysis. Therefore, we conclude that on top of the most central hub *Rictor*, the orchestra of the whole molecular network might be responsible for the cardioprotective effects

## 4. Materials and Methods

### 4.1. In Silico Analysis of the ProtectomiR–mRNA Target Interaction Network

Potential mRNA targets of 18 different previously discovered and validated mimic and antagomiR protectomiRs [5] were predicted by miRNAtarget™ software developed by our team recently (www.mirnatarget.com; Pharmahungary, Szeged, Hungary; accessed at several days in August, 2017). As in other studies [8,9,10,11], the protectomiR–mRNA target interaction network was created analyzing the Norway rat version of two predicted (miRDB version 5.0; microRNA.org version released in 2010) and one experimentally validated, manually curated (miRTarBase 4.5) microRNA–target interaction databases [27,28,29]. As in the previous studies, the inclusion thresholds for miRDB and mirSVR scores were >80.0 and <−1.2, respectively. In the constructed protectomiR–target interaction network protectomiRs and predicted targets appear as nodes while edges between them represent predicted microRNA–target interactions. To express the number of microRNAs interacting with their predicted mRNA targets node degree (i.e., the number of the incoming edges) was calculated for each mRNA target, and mRNA targets were sorted in the descending order of the node degrees. Target nodes with the highest node degree values were considered as microRNA target hubs and were selected for further validation.

The protectomiR–target interaction network was visualized by the EntOptLayout plugin version 2.1 (https://apps.cytoscape.org/apps/EntOptLayout) for the Cytoscape framework (version 3.7.2, Cytoscape Consortium, San Diego, CA, USA) [30]. For the optimization of the network layout double consideration of the main diagonal of the adjacency matrix was chosen and alternating position and width updates were performed.

### 4.2. Cross-Species Rat–Pig microRNA Similarity Matching

The NCBI RNA BLAST and miRBase [31] databases were used to identify pig microRNAs with sequence similarity to the selected rat microRNAs, which were targeting the central mRNA hub of the interaction network.

### 4.3. Porcine Myocardial Tissue Samples

To validate rat Protectomirs and their central target experimentally, myocardial tissue samples were obtained from a previously published, well characterized, clinically relevant, closed-chest porcine model of reperfused acute myocardial infarction and cardioprotection [13]. In this model, ischemic preconditioning is considered as the gold standard cardioprotective intervention regarding to the infarct size reducing effect. In postconditioning, oedema and microvascular obstruction was reduced as signs of vascular protection (for more details on the measured parameters, see reference [13]). Tissue samples of the following groups were used in the present study for PCR and western blots:

- Ischemia-reperfusion group (Isch): 90 min myocardial ischemia was induced with the balloon occlusion of the left anterior descending (LAD) coronary artery followed by 3 h of reperfusion (*n* = 6, except for ischemic zone western blot *n* = 4. Difference in group size was due to availability of samples).
- Ischemic preconditioned group (IPreC): 3 × 5 min myocardial ischemia was applied before the 90 min LAD occlusion followed by 3 h of reperfusion (*n* = 4, except for HSP western blots *n* = 3, difference in group size was due to availability of samples).
- Ischemic postconditioned group (IPostC): 6 × 30 s myocardial ischemia was applied after the 90 min LAD occlusion, at the start of the 3 h reperfusion (*n* = 6, except for ischemic zone western blots *n* = 4. difference in group size was due to availability of samples).

Animals were sacrificed and myocardial tissue samples were collected from both the ischemic and non-ischemic remote regions of the left ventricle in each groups after a 3 h reperfusion (for further details, see reference [13]). Samples were snap-frozen in liquid nitrogen immediately and stored at −80 °C.

### 4.4. Total RNA Isolation

Total RNA was isolated from left ventricular samples (*n* = 4–6/group) with a Zymo Direct-zol RNA Miniprep kit (Zymo Research, Irvine, CA, USA; Cat. No #R2050) following the manufacturer instructions. DNase I (Thermo Fischer Scientific, Waltham, MA, USA; Cat. No. #EN0521) treatment was applied after total RNA elution in RNase-free water. RNA concentration was determined by spectrophotometry (NanoDrop, Thermo Fischer Scientific, Waltham, MA, USA).

### 4.5. microRNA cDNA Synthesis, and qRT-PCR

cDNA was synthesized from 10 ng total RNA using a miRCURY LNA RT kit (Qiagen, Hilden, Germany; Cat. No. #339340) according to the manufacturer’s protocol. cDNA was further diluted 60× with RNase-free water. qRT-PCR reactions were performed on a LightCycler^®^ 480 II instrument (Roche, Penzberg, Germany) by using a miRCURY LNA SYBR Green PCR kit (Qiagen, Hilden, Germany; Cat. No. #339345). Polymerase was heat-activated for 2 min at 95 °C, and targets were amplified and quantified in 45 cycles (denaturation: 10 s at 95 °C; combined annealing/synthesis: 60 s at 56 °C).

Forward and reverse primers for the ssc-miR-139-5p (Qiagen, Hilden, Germany; Cat. No. YP00204037), ssc-miR-212 (Cat. No. YP00205401), ssc-miR-320 (Cat. No. YP02114214), ssc-miR-362 (Cat. No. YP02113056) and ssc-miR-503-5p (Cat. No. YP00205094) were used for analysis. U6 snRNA (Cat. No. YP00203907) was used as a housekeeping gene. Results were calculated with the 2^−ΔΔCp^ evaluation method.

### 4.6. mRNA cDNA Synthesis, and qRT-PCR

cDNA was synthesized from 1 µg total RNA using a Sensifast cDNA synthesis kit (Bioline, London, UK; Cat. No. #BIO-65053) according to the manufacturer’s protocol. cDNA was further diluted 20× with RNAse-free water. qRT-PCR reactions were performed on a LightCycler^®^ 480 II instrument (Roche, Penzberg, Germany) by using the SensiFAST SYBR Green master mix (Bioline, London, UK; Cat No. #BIO-98005). Polymerase was heat-activated for 2 min at 95 °C, and targets were amplified and quantified in 40 cycles (denaturation: 5 s at 93 °C; annealing: 10 s at 60 °C; synthesis: 20 s at 72 °C).

Forward and reverse primers for the RPTOR independent companion of MTOR complex 2 (*Rictor*) were used for analysis. Beta-actin (*Actb*) was used as a housekeeping gene. Results were calculated with the 2^−ΔΔCp^ evaluation method. Sequences of primers are shown in Supplementary Figure S2.

### 4.7. Western Blots

Snap frozen heart samples were homogenized in radioimmunoprecipitation assay buffer (Cell Signaling Technology, Danvers, MA, USA) containing protease inhibitor cocktail (complete EDTA-free ULTRA Tablets, Roche, Germany; phenylmethylsulfonyl fluoride, Sigma, St. Louis MO, USA). Protein concentration was measured with a bicinchoninic acid assay (Thermo Fischer Scientific, Waltham, MA, USA). Equal amounts of protein were mixed with Laemmli buffer, and were separated in 4–15% Mini-PROTEAN^®^ TGX™ Gel (Biorad, Hercules, CA, USA). Proteins were transferred onto a polyvinylidene difluoride membrane (Biorad, Hercules, CA, USA). The membrane was stained for quantification of total protein expression with Mem-Code (Thermo Fischer Scientific, Waltham, MA, USA, Cat. No. #24580) total protein stain according to the manufacturer’s description. The membrane was blocked with Blotting-Grade Blocker (Biorad, Hercules, CA, USA). Membranes were incubated with primary antibodies (anti-RICTOR, Abcam, Cambridge, UK; Cat. No. #ab105469, anti-GAPDH, Cell Signaling Technology, Danvers, MA, USA; Cat. No. #5174, anti-ACTB, Cell Signaling Technology, Danvers, MA, USA; Cat. No. #12620, anti-tubulin, Abcam, Cambridge, UK; Cat. No. #ab6046, anti-phospho PKC and total PKC Cell Signaling, Danvers, MA, USA, Cat. No. #38938 and #2056, anti-phospho Akt and total Akt, Cell Signaling, Danvers, MA, US, Cat. No. #9271, #9271, anti-phospho GSK-3, Cell Signaling, Danvers, MA, USA, Cat. No.#9336 and #9315, anti-HSP70 Santa Cruz Biotechnology, Dallas, TX, USA, Cat. No. sc-32239, HSP-90, Santa Cruz Biotechnology, Dallas, TX, US, Cat. No. sc-13119, HSP data were normalized to anti-ACTB, Santa Cruz Biotechnology, Dallas, TX, USA, Cat. No. sc-130657), and thereafter with corresponding horseradish-peroxidase-conjugated secondary antibodies (Cell Signaling Technology, Danvers, MA, USA or Santa Cruz Biotechnology, Dallas, TX, USA). After incubating the membranes with a 3:7 ratio mix of Clarity Max and Clarity ECL Western Blotting Substrate (Biorad, Hercules, CA, USA; Cat. No. #1705062S and #1705060), proteins of interest were detected with ChemiDoc XRS+ System (Biorad, Hercules, CA, USA). Band densities were analyzed with planimetry and compared to total protein staining. Expression pattern of different control genes is presented in Supplementary Figure S4.

### 4.8. Gene Ontology Enrichment Analysis

An online PANTHER Overrepresentation test (geneontology.org, version released on 11 July 2019 [32]) was used to perform Gene Ontology (GO) biological process enrichment analysis on the full list of 882 ProtectomiR target genes. To adjust for multiple hypothesis testing, Bonferroni correction was applied. In this analysis the version of the GO Ontology Database [33] released on 9 December 2019 was used as a source for *Rattus norvegicus* gene annotations.

## 5. Conclusions

This is the first demonstration that *Rictor* is the central molecular target of ProtectomiRs and that decreased *Rictor* expression may regulate ischemic postconditioning- but not preconditioning-induced acute cardioprotection. We concluded that *Rictor* is a potential drug target for acute cardioprotection.

## Figures and Tables

**Table 1:** Top listed predicted mRNA targets of ProtectomiR microRNAs revealed by interaction network and microRNA–mRNA target prediction analysis. From the total number of mRNAs regulated by up- or downregulated ProtectomiR microRNAs the listed 15 mRNAs had at least three microRNA–mRNA target interactions (node degree). *Rictor*, the target hub, was ranked first, involved in five microRNA–mRNA target interactions (degree 5). (Complete list of predicted target mRNAs is available in the Supplementary Materials (Supplementary Table S1)).

Degree	Symbol	Gene Name
5	*Rictor*	RPTOR independent companion of MTOR, complex 2
3	*Arih1*	Ariadne RBR E3 ubiquitin protein ligase 1
3	*Cd244*	CD244 molecule
3	*Crisp1*	Cysteine-rich secretory protein 1
3	*Ctbs*	Chitobiase
3	*E2f5*	E2F transcription factor 5
3	*Ehhadh*	Enoyl-CoA hydratase and 3-hydroxyacyl CoA dehydrogenase
3	*Ets1*	ETS proto-oncogene 1, transcription factor
3	*Hsd17b2*	Hydroxysteroid (17-beta) dehydrogenase 2
3	*Mapk6*	Mitogen-activated protein kinase 6
3	*Mier3*	Mesoderm induction early response 1, family member 3
3	*Pde12*	Phosphodiesterase 12
3	*Slc20a1*	Solute carrier family 20 member 1
3	*Srsf7*	Serine and arginine rich splicing factor 7
3	*Yod1*	YOD1 deubiquitinase

## Data Availability

Not applicable.

## Supplementary Materials

The following are available online at https://www.mdpi.com/article/10.3390/ijms22179539/s1, Figure S1: High resolution microRNA-target interaction network, Figure S2: Supplementary material for qPCR experiments, Figure S3: Expression and phosphorylation of RICTOR/mTORC2 downstream effectors and interacting proteins, Figure S4: Protein level expression pattern of different control genes, Table S1: List of mRNA targets of ProtectomiR microRNAs, Table S2: GO analysis results.
