# Supplementary figures and images for: Molecular Network Approach Reveals Rictor as a Central Target of Cardiac ProtectomiRs
Source: Int J Mol Sci. 2021 Sep 2;22(17):9539. doi: 10.3390/ijms22179539 (PMC8430799; doi:10.3390/ijms22179539)

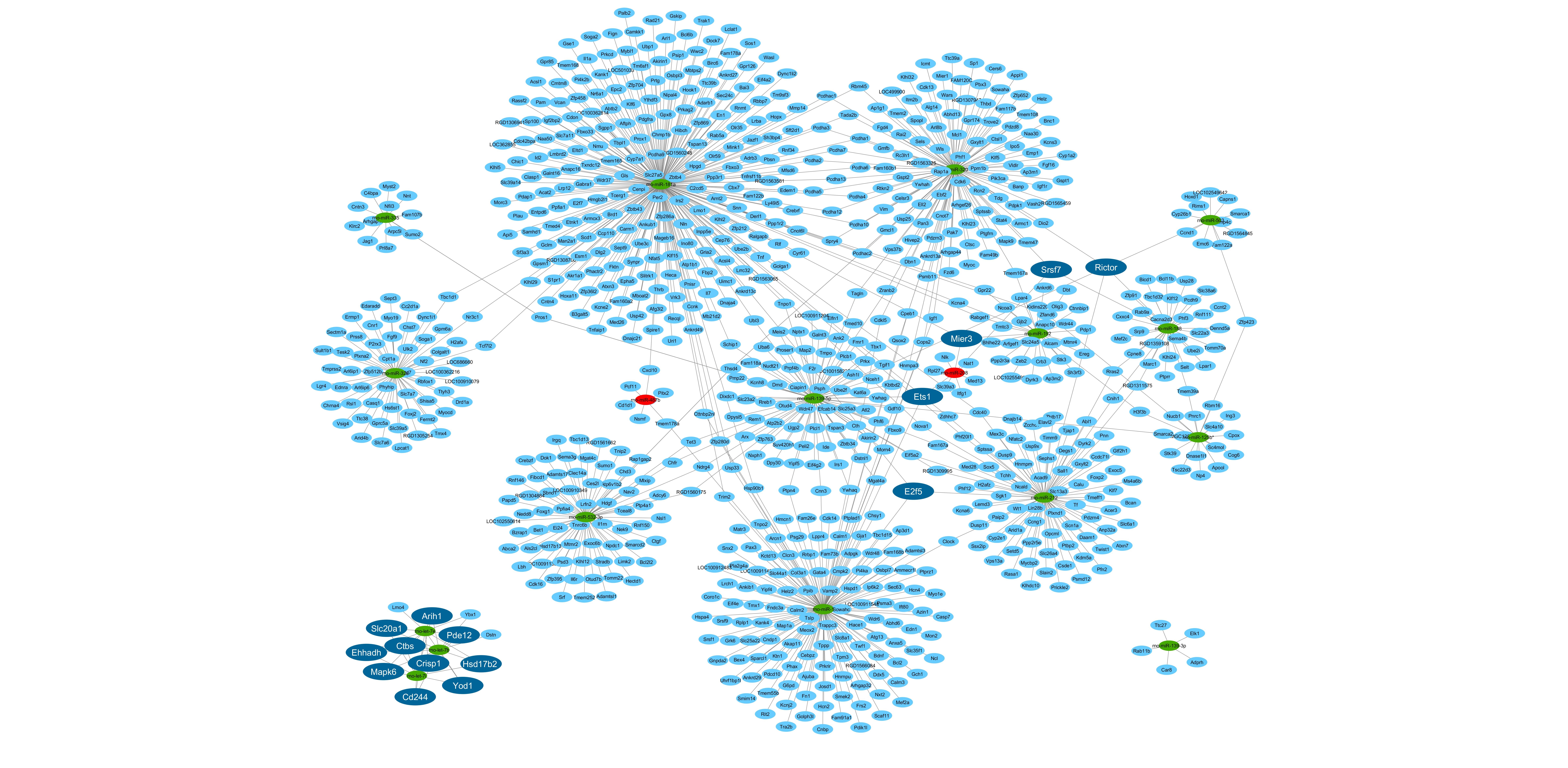

Supplement: Supplementary file 1 [file ijms-22-09539-s001.zip › Supplementary Figure S1_protectomir_full network.jpeg]

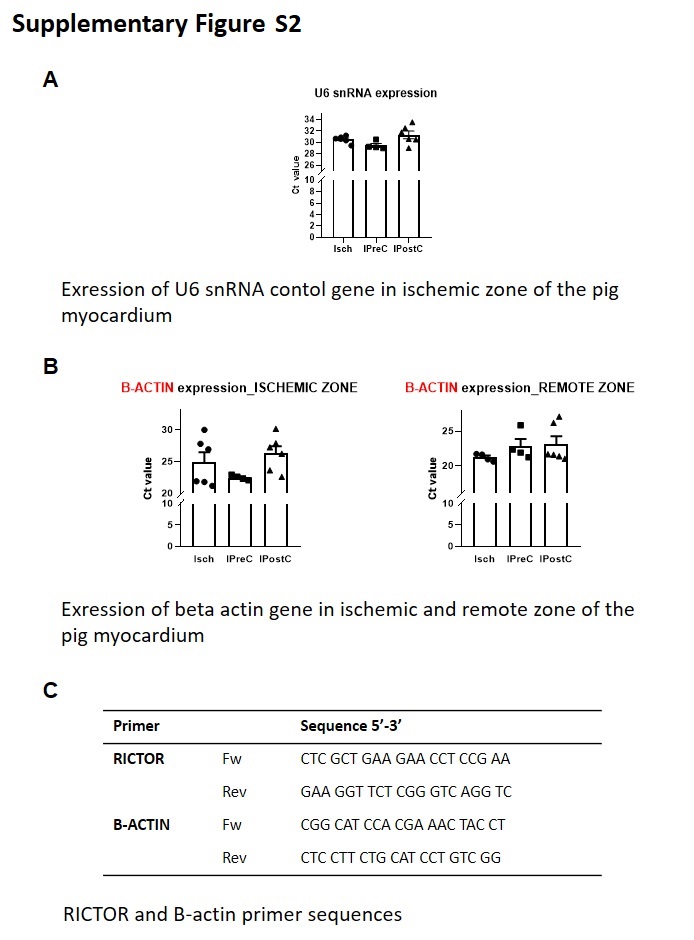

Supplement: Supplementary file 1 [file ijms-22-09539-s001.zip › Supplementary Figure S2.jpg]

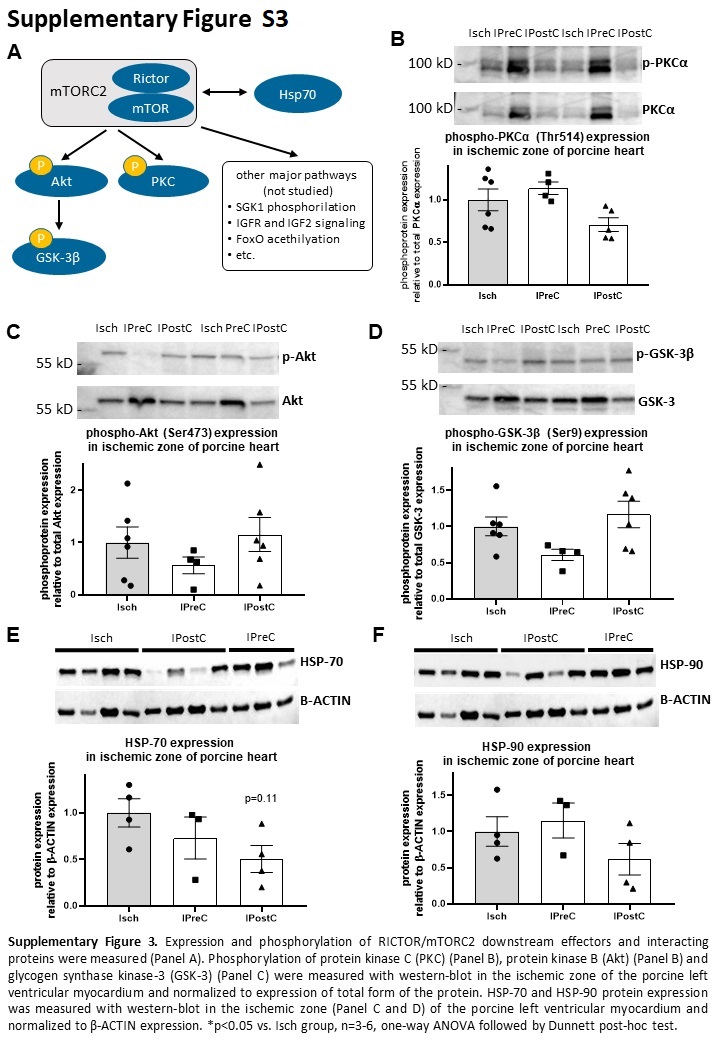

Supplement: Supplementary file 1 [file ijms-22-09539-s001.zip › Supplementary Figure S3 new.jpg]

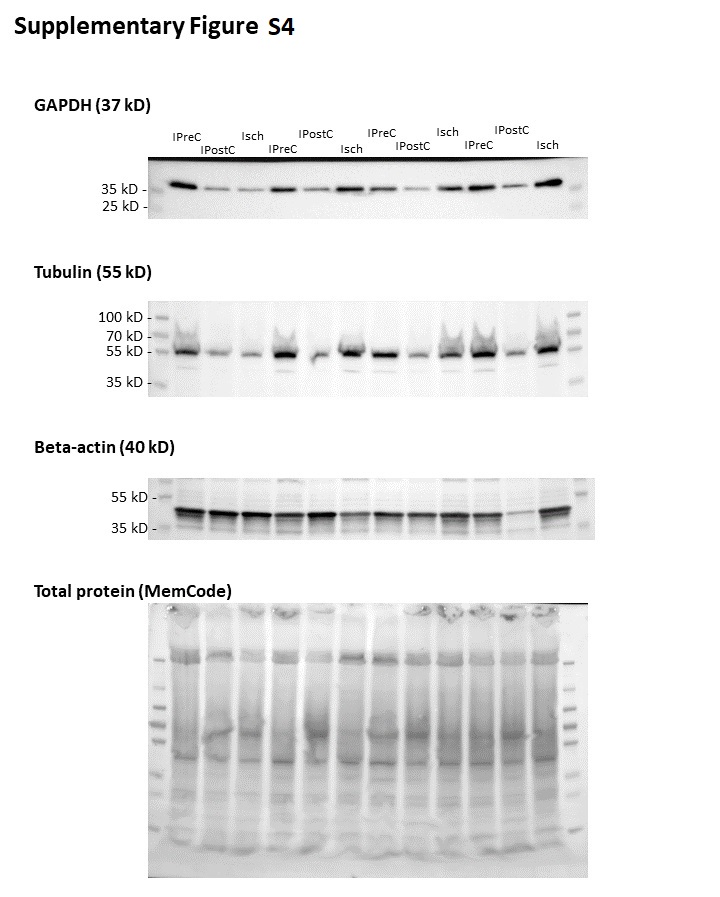

Supplement: Supplementary file 1 [file ijms-22-09539-s001.zip › Supplementary Figure S4 new.jpg]
